# A Novel High-Precision Workpiece Self-Positioning Method for Improving the Convergence Ratio of Optical Components in Magnetorheological Finishing

**DOI:** 10.3390/mi16070730

**Published:** 2025-06-22

**Authors:** Yiang Zhang, Pengxiang Wang, Chaoliang Guan, Meng Liu, Xiaoqiang Peng, Hao Hu

**Affiliations:** 1 College of Intelligence Science and Technology, National University of Defense Technology, Changsha 410073, China; 2 National Key Laboratory of Equipment State Sensing and Smart Support, Changsha 410073, China; 3 Hunan Key Laboratory of Ultra-Precision Machining Technology, Changsha 410073, China

**Keywords:** magnetorheological finishing, optical smart manufacturing, workpiece positioning error, workpiece self-positioning, stepwise global optimization

## Abstract

Magnetorheological finishing is widely used in the high-precision processing of optical components, but due to the influence of multi-source system errors, the convergence of single-pass magnetorheological finishing (MRF) is limited. Although iterative processing can improve the surface accuracy, repeated tool paths tend to deteriorate mid-spatial frequency textures, and for complex surfaces such as aspheres, traditional manual alignment is time-consuming and lacks repeatability, significantly restricting the processing efficiency. To address these issues, firstly, this study systematically analyzes the effect of six-degree-of-freedom positioning errors on convergence behavior, establishes a positioning error-normal contour error transmission model, and obtains a workpiece positioning error tolerance threshold that ensures that the relative convergence ratio is not less than 80%. Further, based on these thresholds, a hybrid self-positioning method combining machine vision and a probing module is proposed. A composite data acquisition method using both a camera and probe is designed, and a stepwise global optimization model is constructed by integrating a synchronous iterative localization algorithm with the Non-dominated Sorting Genetic Algorithm II (NSGA-II). The experimental results show that, compared with the traditional alignment, the proposed method improves the convergence ratio of flat workpieces by 41.9% and reduces the alignment time by 66.7%. For the curved workpiece, the convergence ratio is improved by 25.7%, with an 80% reduction in the alignment time. The proposed method offers both theoretical and practical support for high-precision, high-efficiency MRF and intelligent optical manufacturing.

## 1. Introduction

Magnetorheological finishing (MRF), as a deterministic optical manufacturing method, plays a pivotal role in the fabrication of high-precision optical components such as lithographic objectives, X-ray mirrors, and deep-space exploration reflectors [1,2,3,4,5]. During the polishing process, coupling effects from multiple error sources, such as fluctuations in the removal function (a mathematical model describing material removal per unit processing time), edge effects, and positioning inaccuracies, often lead to significant deviations between the actual and ideal convergence ratios [6] (the ratio of RMS after polishing to initial RMS). Current processes rely on iterative polishing to improve the surface accuracy; however, repeated passes along fixed tool paths tend to degrade mid-spatial frequency textures. Surface figure errors may cease to converge or even diverge after reaching a certain accuracy threshold [7]. For large-aperture aspheres and other complex freeform surfaces, manual alignment and adjustment can take several hours and typically result in poor repositioning precision, severely limiting both the efficiency and accuracy. Effectively suppressing systematic errors and improving the single-pass convergence ratio remains a pressing challenge in ultra-precision optical fabrication.

Regarding the stability of the removal function, studies have shown that variations in the rheological properties of the polishing fluid, immersion depth of the polishing ribbon, and magnetic field fluctuations can lead to spatial non-uniformities in the removal function, thereby inducing mid-spatial frequency (MSF) errors [8,9]. Liu et al. introduced a characteristic length coefficient for the removal function, enabling ripple error suppression through optimization of the coefficient and step size [10]. Wang et al. proposed an optimal single-pass material removal thickness, which effectively mitigated MSF errors caused by removal function instability [11]. Significant progress has also been made in predictive compensation methods based on process parameters and material removal mechanisms [12,13,14]. For instance, Liu developed an integrated pressure–shear force model capable of accurately predicting both the shape and removal rate of the function [15], while Yan et al. designed a distributed neural network model achieving an average prediction accuracy above 95% [16]. For edge effect suppression, Hu et al. experimentally analyzed the variation in removal functions near boundaries and proposed two strategies: applying smaller removal functions at the edges and adjusting dwell time calculations accordingly [17]. Tang et al. improved the Gerchberg algorithm, reducing the edge effect radius from 5 mm to 2 mm and lowering the RMS residual from 19.3 nm to 9.7 nm [18]. Jeon et al. developed a model to predict nonlinear edge effects in MRF based on polishing parameters, with a relative prediction error ranging from 4% to 7% [19]. To suppress convolution effects, increasing the irregularity of polishing paths has proven critical for MSF error reduction [20,21,22]. Zhao et al. proposed a six-directional pseudo-random continuous single-line path, which leveraged multidirectional and highly stochastic motion to significantly reduce MSF errors [23]. Wang et al. introduced the concept of an angle-step state, achieving the effective suppression of MSF errors in both planar and aspheric surfaces [24]. In terms of dwell time computation, various approaches have been extensively explored, including global optimization using particle swarm algorithms [25], non-sequential strategies via genetic algorithms [26], and refined non-negative least squares methods [27]. Considering the limited dynamic performance of CNC systems, Zhang et al. proposed a bounded constrained least squares (BCLS) algorithm that incorporates machine dynamics, resulting in a 55.2% improvement in the machining accuracy [28].

Compared with the extensive research on the aforementioned error sources, the control of workpiece positioning errors remains a technical bottleneck. Zhou et al. improved the iterative closest point (ICP) algorithm using probe-collected data and proposed self-localization methods for square and rotationally symmetric workpieces, achieving a positioning accuracy better than 10 µm [29,30]. Peng et al. developed a synchronous iterative localization algorithm based on probe measurements, which significantly reduced the setup time and improved the polishing convergence ratio [31]. However, both approaches require manual pre-alignment and depend on probe-based edge coordinate acquisition, which suffers from high noise and limited precision. As a result, the stability and efficiency of these localization methods remain suboptimal. In other fields, self-localization technologies based on advanced sensors have developed rapidly [32,33]. de Araujo et al. designed a vision-based self-localization system for CNC milling machines, achieving an absolute positioning accuracy better than 80 µm [34]. Nonetheless, stereo-vision techniques only apply to objects with rich textures and generally offer a limited positioning accuracy. To enhance texture information, researchers have employed structured light projection. For example, Zhou et al. used line-structured light to localize and reconstruct weld seams with an accuracy better than 100 µm [35]. However, structured light projection is limited to diffusely reflective surfaces. Zhang et al. constructed an in situ measurement system based on phase deflectometry for ultra-precision turning machines, which enabled in-place localization and the measurement of workpieces [36]. Despite its potential, this method is constrained by the display screen size, leading to a limited measurement range and insufficient reconstruction accuracy to meet the stringent requirements of MRF.

**Figure 1:**
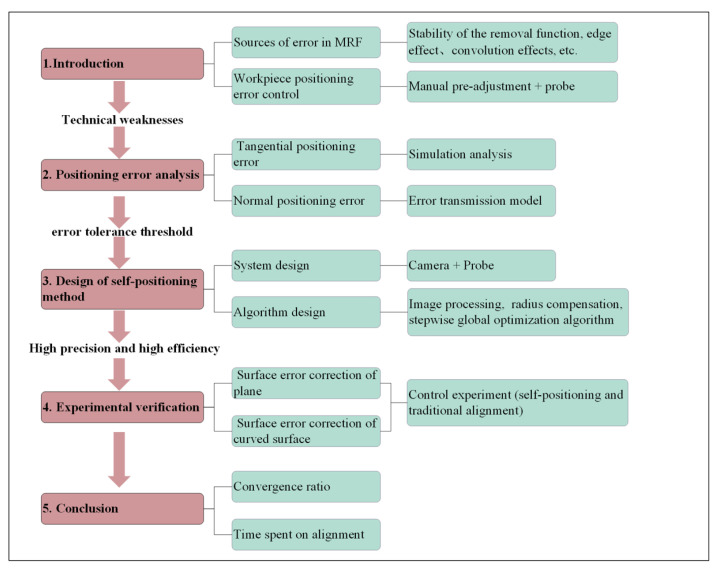
Research framework structure.

In summary, while error sources such as removal function fluctuations and edge effects in magnetorheological finishing have been thoroughly investigated, the research on workpiece positioning errors remains technically deficient, with unclear influence mechanisms. Existing self-positioning methods for workpieces all rely on manual pre-installation and adjustment, and employ probe-based data acquisition, suffering from limitations including low efficiency and being incapable of phase information localization. This study clarifies the influence mechanism of workpiece positioning errors and proposes a vision–probe integrated positioning method, eliminating the need for manual pre-installation, enabling precise phase information localization. Meanwhile, the number of probe measurement points is reduced to 9 (25 points required in reference [31]). The research framework of this paper is illustrated in Figure 1. Firstly, we systematically investigate the impact of six-degree-of-freedom positioning errors on surface figure convergence, establish a positioning error-normal contour error transfer model, and clarify the error tolerance thresholds of each degree of freedom; secondly, we propose a hybrid self-localization method combining machine vision and probe measurements, and construct a stepwise global optimization model, which achieves a positioning accuracy of better than 10 μm/0.005° (100 mm in diameter). Finally, the proposed self-positioning method is successfully applied to the engineering machining of planar and spherical surfaces, which improves the convergence ratio of the magnetorheological finishing error by 25–40%, and greatly shortens the setup time simultaneously.

## 2. Influence of Workpiece Positioning Errors on Polishing Convergence Ratio

In the magnetorheological finishing process, the existence of workpiece positioning errors can cause the actual convergence ratio to be significantly lower than the theoretical value. In order to reveal the intrinsic mechanism of this phenomenon, numerical simulation is used to systematically study the influence law of the positioning error of each degree of freedom on the surface figure convergence process. The relative convergence ratio of 80% (the ratio of the actual convergence ratio to the ideal convergence ratio) is used as the evaluation index to determine the error tolerance threshold of each degree of freedom.

### 2.1. Sources of Positioning Errors

Magnetorheological finishing (MRF) is a deterministic sub-aperture polishing method based on the computer-controlled optical surfacing (CCOS) principle. Its core mechanism involves the precise removal of surface figure errors guided by quantitatively acquired measurement data. As expressed in Equation (1), the material removal volume H(x,y) of an optical component is mathematically characterized by the convolution of the tool’s removal function R(x,y) and the dwell time distribution T(x,y) along the planned polishing path [6]:

(1) H(x,y)=R(x,y)∗T(x,y)

**Figure 2:**
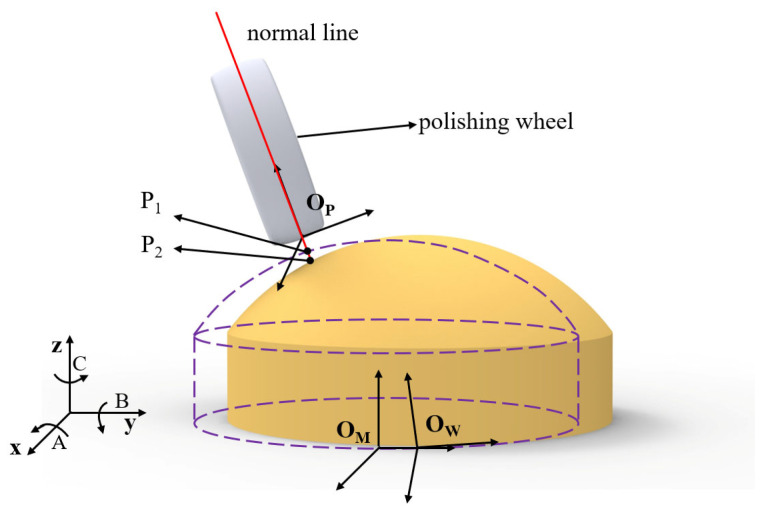
The relationships among workpiece coordinate system (O_W_-_XYZ_), program coordinate system (O_M_-_XYZ_), and polishing tool coordinate system (O_P_-_XYZ_).

The key to achieving deterministic correction lies in two aspects: precise localization of the machining point and accurate control of dwell time. As illustrated in Figure 2, three coordinate systems are involved in the MRF process: the workpiece coordinate system (O_W_-_XYZ_), the program coordinate system (O_M_-_XYZ_), and the polishing tool coordinate system (O_P_-_XYZ_). Both the dwell time calculation and tool path generation are based on the workpiece coordinate system. However, during actual machining, alignment between the workpiece and program coordinate systems is typically adjusted manually using a percent meter. This process inevitably introduces positioning errors:

(2) $$P_{W}=T^{-1}\cdot P_{M}$$

where **P***_W_* represents the coordinates in the workpiece coordinate system, while **P***_M_* denotes the corresponding point in the program coordinate system. A six-degree-of-freedom rigid transformation matrix **T** relates the two systems. The presence of **T** introduces spatial deviations from the ideal material removal path, resulting in reduced polishing convergence efficiency. The following sections analyze the influence of errors in each of the six degrees of freedom (X, Y, Z, A, B, and C) on the convergence ratio.

### 2.2. Tangential Positioning Error

Previous studies [6] have demonstrated that variations in the indentation depth significantly affect both the shape of the removal function and the material removal efficiency. Based on whether the shape of the removal function changes, positioning errors can be categorized as tangential or normal errors. For planar workpieces, translation errors along the X and Y axes and rotational errors around the C axis are considered tangential, while translation along the Z axis and rotations around the A and B axes are classified as normal errors. However, for rotationally symmetric surfaces, only the C-axis rotational error constitutes a tangential error; all other degrees of freedom contribute to normal errors.

For a planar surface, let the positioning errors in the X and Y directions be denoted by  ∆x,∆y, respectively. The material removal volume can then be expressed as

(3) $$H_{1}(x,y)=R(x-△x,y-△y)*T(x,y)$$

(4) $$error_{1}(x,y)=H(x,y)-H_{1}(x,y)$$

where  R(x−∆x,y−∆y) is the removal function considering positioning errors, and T(x,y) represents the dwell time under ideal conditions. $H_{1}(x,y)$ denotes the actual material removal, and ${error}_{1}\left(x,y\right)\;$ is the resulting surface error after machining.

For a rotational error ∆c around the *C* axis, the removal function with error $R\prime (x^{\prime },y\prime )$ can be described as

(5) $${\left[\begin{array}{ccc} x\prime & y\prime & R\prime (x\prime ,y\prime ) \end{array}\right]}^{T}=T_{3\times 3}\cdot {\left[\begin{array}{ccc} x & y & R(x,y) \end{array}\right]}^{T}$$

where **T**_3×3_ is the rotation matrix. Similarly, the surface error after machining ${error}_{2}(x,y)$ is given by

(6) $$error_{2}(x,y)=H(x,y)-H_{2}(x,y)$$

Since both the initial surface error H(x,y) and the removal function $R\left(x,y\right)\;$ are represented as discrete data, analytical expressions are difficult to obtain. Therefore, a simulation-based method is adopted to investigate the influence of positioning errors on the polishing performance. The relative convergence ratio η is used as the evaluation metric, defined as follows:

(7) $$\eta =\frac{RMS_{idea1}}{RMS_{actua1}}\times 100\%$$

where RMS_actual_ represents the actual surface error after machining, while RMS_ideal_ denotes the ideal surface error following processing.

**Figure 3:**
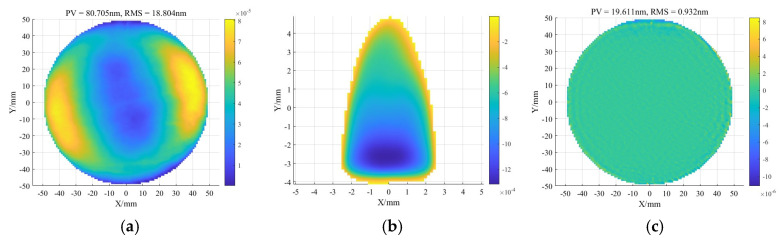
Initial conditions of simulation: (**a**) Initial surface figure error. (**b**) Removal function. (**c**) Ideal surface figure error.

As shown in Figure 3, for the initial surface figure error and removal function, the surface figure error after polishing is RMS 0.9 nm in the ideal case. Four groups of X-direction positioning errors were introduced, with base values of 0.1 mm, 0.5 mm, 1 mm, and 1.5 mm, each varied by ±10%. The simulation results are presented in Figure 3c. A clear increase in the residual RMS error is observed as the positioning error grows. When the X-direction error reaches 400 μm, the relative convergence ratio drops to 66.95%.

**Figure 4:**
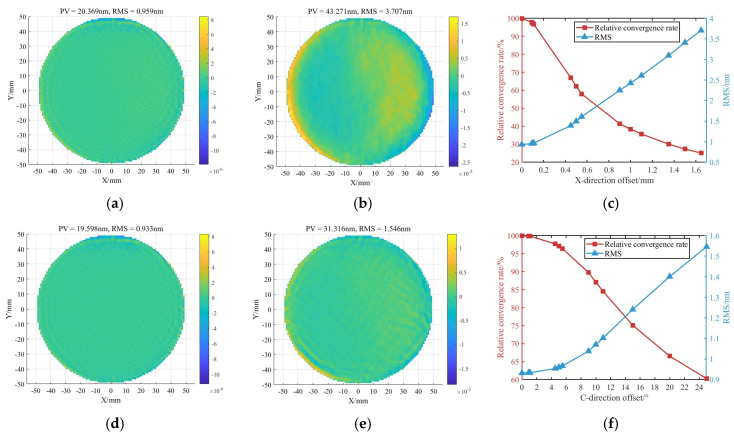
Surface figure simulation results under different tangential errors: (**a**) X-direction offset of 0.1 mm. (**b**) X-direction offset of 1.65 mm. (**c**) Residual errors under various X-direction offsets. (**d**) C-direction rotational error of 1°. (**e**) C-direction rotational error of 25°. (**f**) Residual errors under various C-direction rotational errors.

Three groups of C-direction rotational errors were introduced, with base values of 1°, 5°, and 10°, each varied by ±10%. An additional group was set with a nominal value of 20° and a variation of ±5%. The simulation results are shown in Figure 4f. Compared to errors in the X and Y directions, the influence of C-direction rotational errors is significantly smaller. Even at a rotational error of 10°, the relative convergence ratio remains as high as 87.10%.

### 2.3. Normal Positioning Error

**Figure 5:**
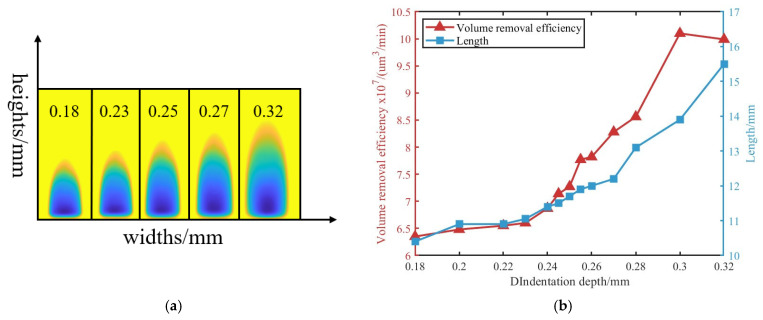
Removal functions at different indentation depths: (**a**) Contour profiles of removal functions at different indentation depths. (**b**) Volume removal efficiency and length under different indentation depths.

Normal positioning errors lead to variations in the shape of the removal function, which are difficult to predict directly. To address this, removal functions obtained under different indentation depths were used to indirectly characterize the effect of normal positioning errors. For a planar surface, removal functions were experimentally acquired under varying indentation depths. The reference indentation depth was set to 0.25 mm, with the detailed experimental parameters listed in Table 1 and the corresponding removal functions shown in Figure 5. When the indentation depth differed by 20 μm, a length deviation of 4% and a volumetric removal efficiency deviation of 13.9% were observed. These results indicate that the indentation depth has a substantial influence on the volumetric efficiency of the removal function.

**Figure 6:**
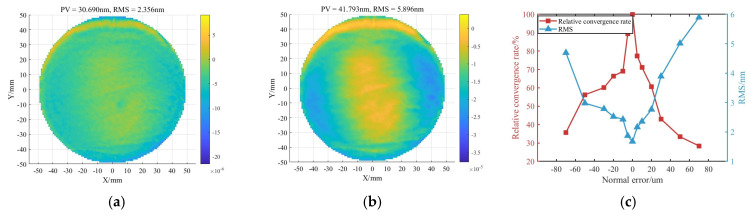
Surface figure simulation results under different normal errors: (**a**) Normal error of 10 μm. (**b**) Normal error of 70 μm. (**c**) Residual surface error under different normal errors.

Taking a planar workpiece as an example, the surface simulation results are shown in Figure 6. As the normal positioning error increases, the relative convergence ratio decreases significantly, reaching 68.99% at an error of 20 μm. The results indicate that normal error is the dominant factor affecting polishing convergence. In addition, positive errors (increased indentation depth) exert a greater impact than negative errors, due to the simultaneous enlargement of the removal area and enhancement of shear force, which together lead to a marked increase in the material removal efficiency.

For curved workpieces, only the C-direction positioning error contributes purely to tangential deviation, while errors in all other directions result from a combined effect of both tangential and normal components. Since both normal errors and curvature variations distort the removal function, directly analyzing the influence of positioning errors on curved surface polishing becomes challenging. Given that tangential errors have a relatively minor impact compared to normal errors, the effect of positioning errors on the polishing convergence is characterized by analyzing the corresponding normal errors.

As shown in Figure 1, during the machining process, the ideal tool point is denoted as **P**_1_, while the actual tool point lies along the tool axis and intersects the workpiece at point **P**_2_. The normal deviation error can thus be expressed as

(8) $$△n=\left\|P_{1}-P_{2}\right\|$$

For an arbitrary surface:

(9) z=f(x,y)

The position and direction vector of the ideal tool point are defined as

(10) $$P_{1}=[a,b,c],u=[u_{x},u_{y},u_{z}]$$

Let L be the line along **u**, and S be the surface; then,

(11) $$\begin{array}{l} L=\{p\in R^{3}|p=p_{0}+tu,t\in R\}\\ S=\{p\in R^{3}|z-f(x,y)=0\} \end{array}$$

The intersection point is given by

(12) $$L\cap S=\{p\in R^{3}|p=p_{0}+tu,z-f(x,y)=0,t\in R\}$$

Due to the presence of positioning error **T**, the surface shape changes in the O_M_ coordinate system, and the actual intersection point becomes

(13) $$L\cap (TS)=T((T^{-1}L)\cap S)$$

The line passing through **P**_1_ can be described as

(14) $$\begin{array}{l} x=a+tu_{x}\\ y=b+tu_{y}\\ z=c+tu_{z} \end{array}$$

After applying the inverse transformation **T**^−1^, the coordinates are transformed as

(15) $$[x\prime ,y\prime ,z\prime ,1]\prime =T^{-1}[x,y,z,1]\prime$$

By substituting $\left[x^{\prime },y^{\prime },z\prime \right]$ into the surface Equation (9), the parameter t can be solved. Let the solution be t_0_; then,

(16) $$P_{2}=[x_{1},y_{1},z_{1},1]=T[a+t_{0}u_{x},b+t_{0}u_{y},c+t_{0}u_{z},1]\prime$$

(17) $$\begin{array}{l} △n=\sqrt{{\left(a-x_{1}\right)}^{2}+{\left(b-y_{1}\right)}^{2}+{\left(c-z_{1}\right)}^{2}}(c>z_{1})\\ △n=-\sqrt{{\left(a-x_{1}\right)}^{2}+{\left(b-y_{1}\right)}^{2}+{\left(c-z_{1}\right)}^{2}}(c<z_{1}) \end{array}$$

Using a spherical mirror with an aperture of 100 mm and a radius of curvature of 400 mm as an example, normal contour error simulations were conducted under various positioning errors, which are listed in Table 2.

**Figure 7:**
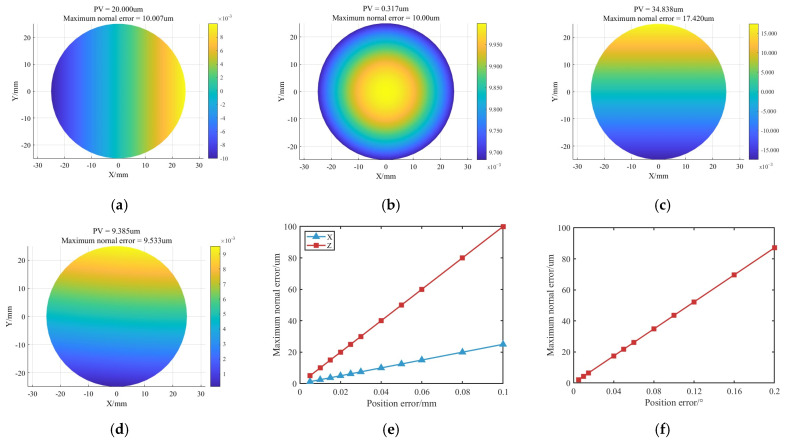
Normal contour errors induced by positioning errors: (**a**) X-direction offset of 0.04 mm. (**b**) Z-direction offset of 0.01 mm. (**c**) A-direction rotational error of 0.04°. (**d**) Combined error. (**e**) Influence of X/Z direction errors on normal contour error. (**f**) Influence of A/B direction errors on normal contour error.

As shown in Figure 7e,f, the maximum normal contour error increases linearly with the magnitude of positioning errors. Among all directions, the Z-direction error produces the largest normal deviation, reaching 20 μm when the positioning error is 20 μm. A/B-direction rotational errors have a secondary impact, with a maximum normal error of 20 μm occurring at a positioning error of 0.04°. X/Y-direction errors exert the least influence, requiring a positioning deviation of 80 μm to reach the same level of normal error. These results indicate that normal errors, particularly those in the Z direction, have a significantly greater effect on polishing convergence than tangential errors, with the C-axis rotational error showing a minimal impact. To ensure polishing accuracy, priority should be given to controlling positioning precision in the Z and A/B directions.

As illustrated in Figure 7d, when the X/Y-direction error is 10 μm, the Z-direction error is 5 μm, and the A/B-direction rotational error is 0.005°, the maximum normal deviation is limited to 9.53 μm, and the relative convergence ratio is not less than 80%. Therefore, the above error value is considered as the tolerance threshold of each degree-of-freedom error (the C-direction error threshold is set to 0.01°), and the relative convergence ratio can be guaranteed to be not less than 80% when the error of each degree of freedom is lower than its corresponding threshold, which, in turn, can effectively inhibit the influence of the localization error on the convergence ratio.

## 3. High-Precision Self-Positioning Method Design

Based on the error tolerance threshold determined above, the design of the self-positioning method is carried out. The vision–probe composite sensing technology is adopted, the image processing algorithm is designed with the optical surface as the positioning reference, and the stepwise global optimization model is constructed to realize high-precision and high-efficiency workpiece self-positioning and ensure that the relative convergence ratio of magnetorheological finishing can be stably maintained at more than 80%.

### 3.1. Overall Design of the Self-Positioning Method

**Figure 8:**
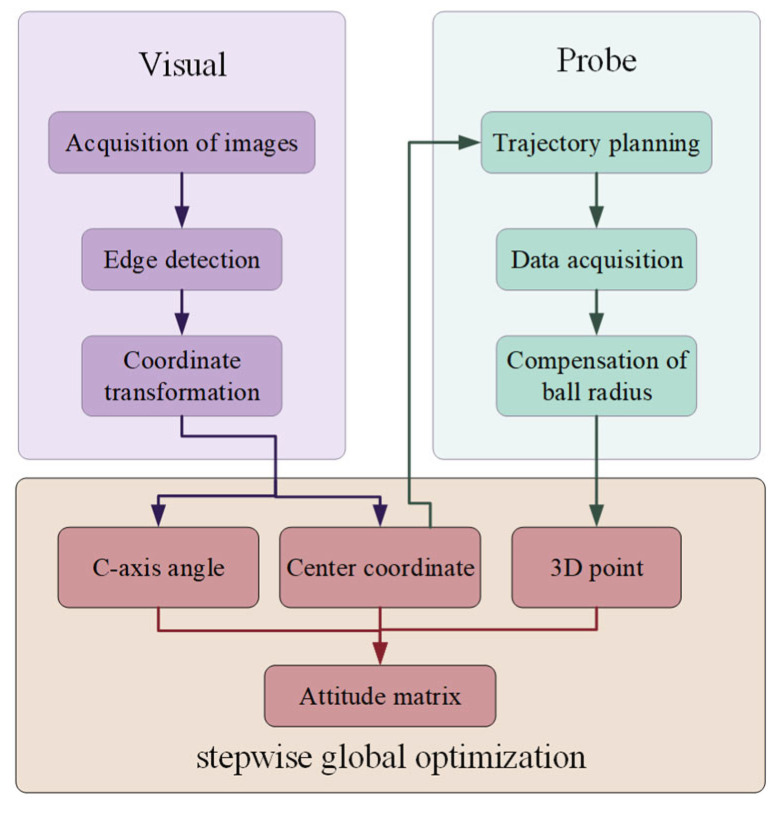
Schematic diagram of the self-positioning method.

As illustrated in Figure 8, the method integrates vision- and probe-based sensing. The vision module acquires surface data to extract two-dimensional edge points. Through edge detection and coordinate transformation, the workpiece center and C-axis rotation angle are calculated. Based on the vision-guided rough positioning, the probe module collects three-dimensional surface data along the surface normal, with compensation applied for the probe tip radius. By integrating the acquired data with the ideal surface model of the workpiece, an optimized pose matrix is obtained to achieve accurate alignment.

**Figure 9:**
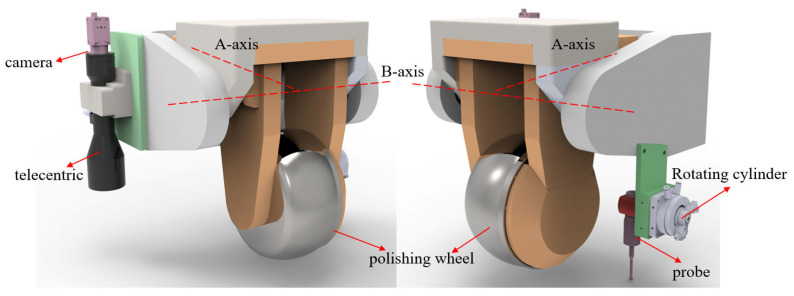
Structural layout of the self-positioning system.

The structural layout of the positioning system is shown in Figure 9. Both the camera and the probe are mounted on the A-axis of the polishing machine. The vision module is fixed in place, while the probe is actuated by a rotary cylinder to switch between measurement and machining positions. The design is compact and ensures collision-free operation. The key performance parameters of the system components are summarized in Table 3.

### 3.2. Vision-Based Center Localization Method

To address the trade-off between field-of-view and measurement accuracy in visual inspection, an image acquisition approach based on CNC machine servo feedback is proposed. The servo system drives the industrial camera to capture multiple edge images while synchronously recording the corresponding machine tool coordinates. Through coordinate transformation, the edge points are mapped to the machine coordinate system. A high-precision, low-distortion telecentric lens is employed in the vision module to ensure measurement accuracy. Given the optical characteristics of the system, a simple nine-point calibration method is sufficient to meet the required precision. The specific calibration model is defined as follows [37]:

(18) $$\left[\begin{array}{c} x\\ y \end{array}\right]=\left[\begin{array}{c} \begin{array}{ccc} a & b & c \end{array}\\ \begin{array}{ccc} d & e & f \end{array} \end{array}\right]\left[\begin{array}{c} u-u_{0}\\ v-v_{0} \end{array}\right]+\left[\begin{array}{c} x_{M}\\ y_{M} \end{array}\right]$$

where $\left(x_{M},y_{M}\right)$ represents the machine tool coordinates recorded during image acquisition; $\left(u_{0},v_{0}\right)$ and (u,v) denote the pixel coordinates of the image center and the detected edge points, respectively. The parameters a, b, c, d, e, and f are calibration coefficients to be determined. (x,y) refers to the machine tool coordinates of the workpiece edge. During the calibration process, nine sets of pixel coordinates and their corresponding machine coordinates are collected. The calibration can be completed by solving a least-squares optimization problem.

**Figure 10:**
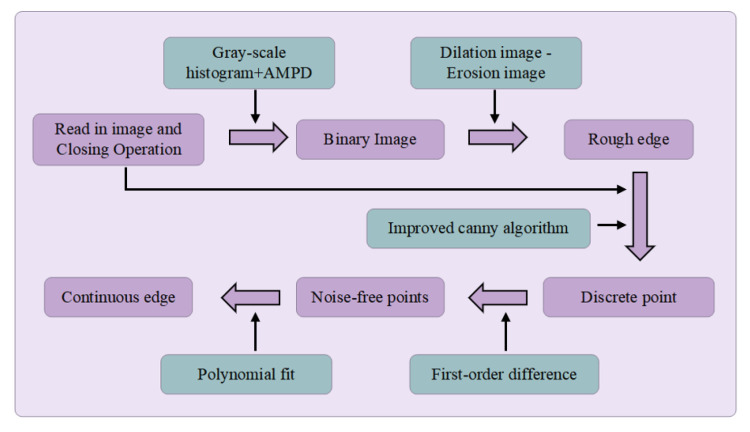
Image processing flowchart.

**Figure 11:**
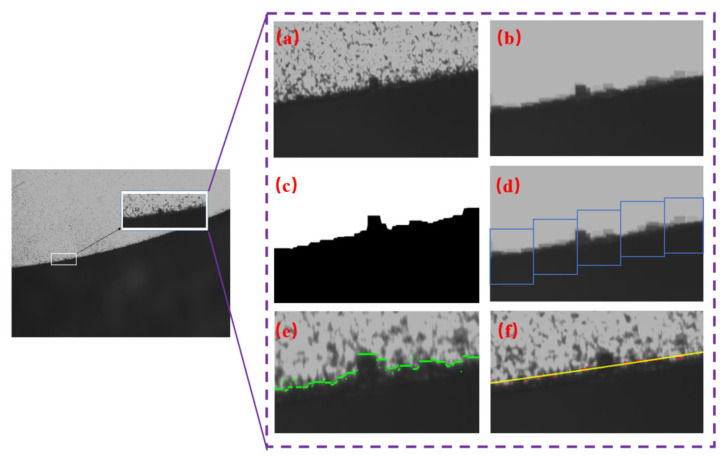
Image processing flowchart: (**a**) Original image. (**b**) Closed operation image. (**c**) Binary image. (**d**) ROI segmentation. (**e**) Edge detection. (**f**) Polynomial fitting of edges.

**Figure 12:**
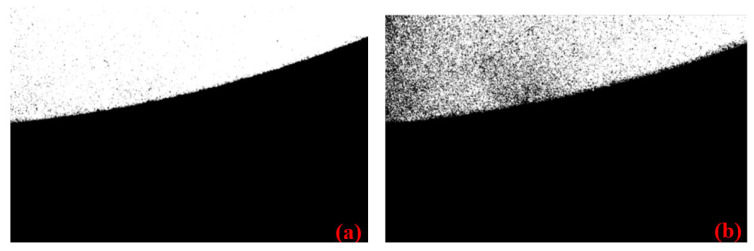
Comparison of threshold segmentation methods: (**a**) Result of triangle thresholding method. (**b**) Result of Otsu’s method.

Taking a circular workpiece as an example, the image processing workflow is illustrated in Figure 10. First, to suppress random noise, such as dark or bright spots in the original image (Figure 11a), a morphological closing operation is applied to remove noise while preserving edge information. Then, image binarization is performed to enhance the contrast between the workpiece and the background, where the choice of threshold is critical. Among existing automatic thresholding methods, the simple thresholding approach relies on manual selection, resulting in low stability and efficiency. Otsu’s method [38] and the triangle algorithm [39] tend to degrade edge integrity, as demonstrated in Figure 12.

**Figure 13:**
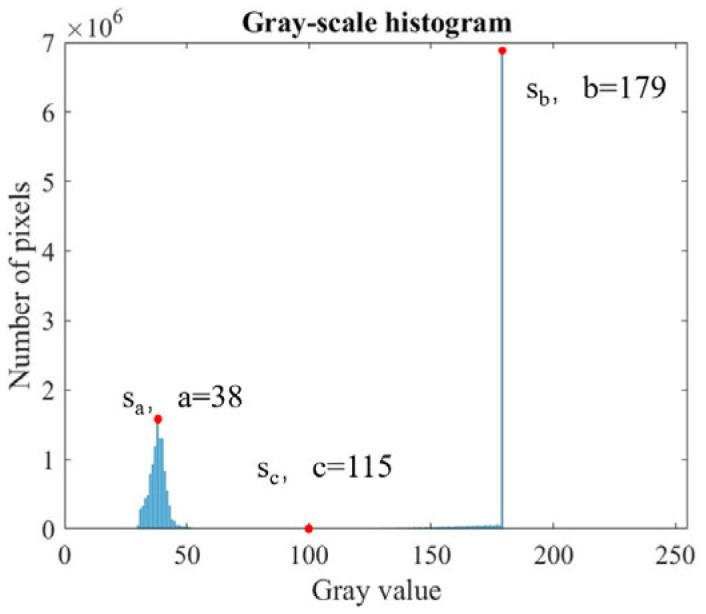
Grayscale histogram after closing operation.

The analysis of the grayscale histogram of the image after morphological closing (Figure 13) reveals a distinct bimodal distribution, indicating the suitability of a simple thresholding approach. To enhance the stability and efficiency of valley detection, the AMPD algorithm [40] is adopted for peak detection and automatic threshold determination. Let the grayscale histogram be represented by the vector $S=[s_{1},s_{2}\ldots s_{255}]$. Using the AMPD algorithm, two prominent peaks can be identified:

(19) $$\left[s_{a},s_{b}]=AMPD(S\right)$$

Let a and b represent the grayscale values corresponding to the two peaks, with a < b. The grayscale value at the valley point is then given by

(20) $$s_{c}=AMPD(-[s_{a},s_{a+1},s_{a+2}\ldots s_{b}])$$

The final result is shown in Figure 13. After threshold calculation, binarization is applied to the image (Figure 11c). The difference between the results of dilation and erosion is used to extract edge contours with pixel-level precision. By segmenting the image along the extracted edge into smaller sub-images, edge detection can be performed locally, which reduces noise and improves the computational efficiency.

**Figure 14:**
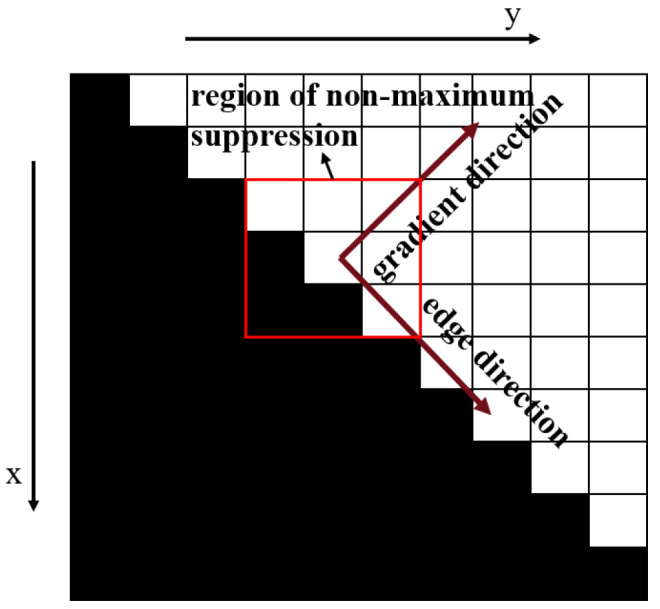
Schematic diagram of edge detection via Canny algorithm.

**Figure 15:**
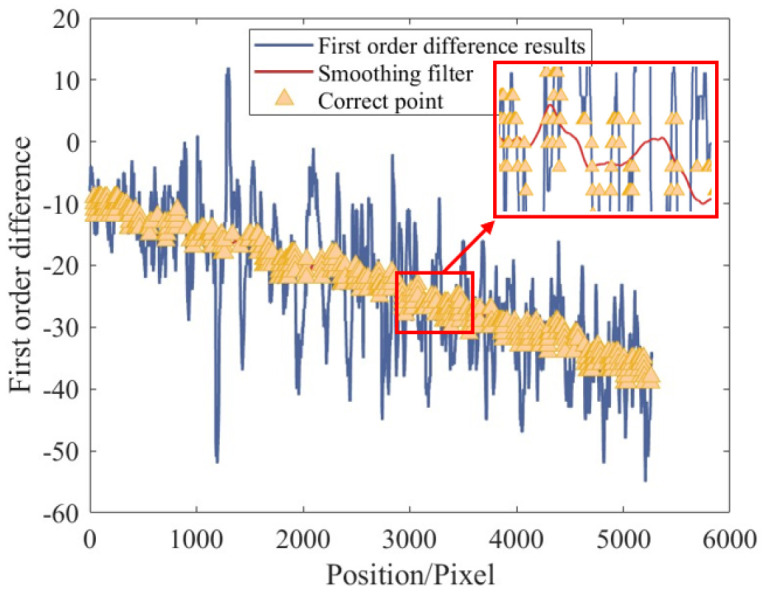
First-order difference performed on detected edges.

As shown in Figure 14, the traditional Canny algorithm [41] performs non-maximum suppression within a 3 × 3 neighborhood along the gradient direction, followed by dual-threshold processing and edge linking. The final result heavily depends on the choice of these thresholds. In contrast, the proposed method extends the non-maximum suppression range to the entire sub-image and eliminates the dual-thresholding and edge-linking steps. This process significantly reduces the dependence on input parameters and produces discontinuous single-sided edges (Figure 11e). To further refine the result, edge continuity is exploited using first-order differences to remove points with abrupt slope changes (Figure 15). Based on the smoothed curve (red line), candidate edge points are selected within a ±2 pixel range. Polynomial fitting is applied to these points to generate a continuous edge contour (yellow curve), as shown in Figure 11f.

Two images are captured along the *x*-axis and *y*-axis of the machine tool, respectively. After image processing and coordinate transformation, four sets of edge coordinate points are obtained and denoted as follows: $N_{i}\left[\left(x_{i1},y_{i1}\right),\left(x_{i2},y_{i2}\right)\ldots \left(x_{in},y_{in}\right)\right]$. Then, for a circular workpiece, the center coordinates $\left(x_{c},y_{c}\right)$ can be calculated as

(21) $$x_{0}=\frac{1}{2n}\sum_{i=1}^{n}\left(x_{1i}+x_{2i}\right)$$

(22) $$y_{0}=\frac{1}{2m}\sum_{j=1}^{m}\left(y_{3j}+y_{4j}\right)$$

**Figure 16:**
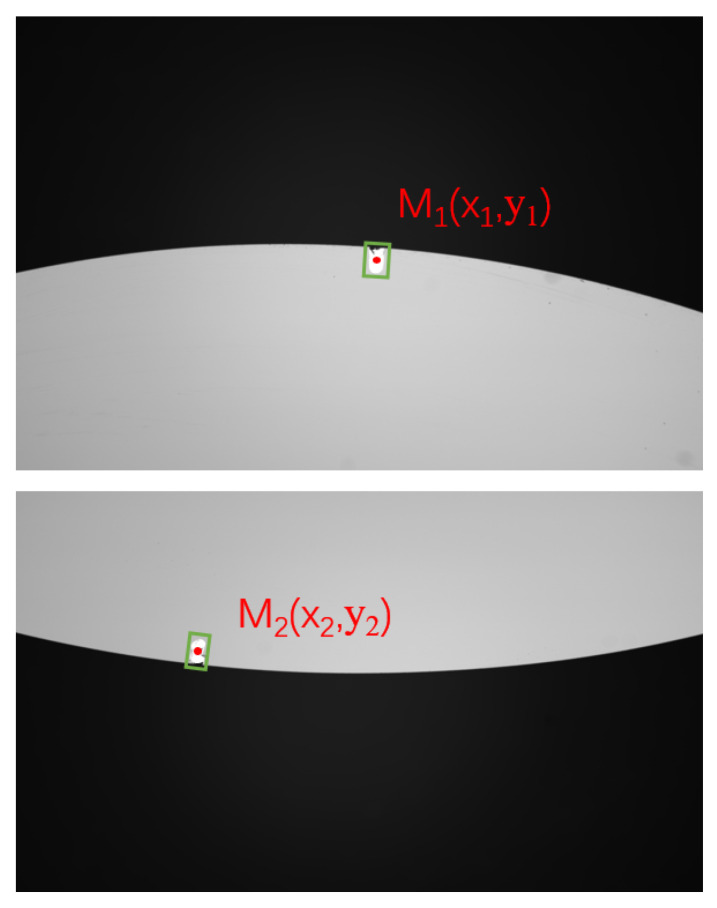
Marker point detection for C-axis positioning.

As shown in Figure 16, two images are captured along the *x*-axis. Using rectangle detection, the reference markers used during interferometric measurement can be identified. Let the center coordinates of the detected rectangles be denoted as $M_{1}(x_{1},y_{1})$ and $M_{2}(x_{2},y_{2})$. The rotation angle around the *C*-axis is then calculated as

(23) $$c_{0}=\arctan(\frac{y_{1}-y_{2}}{x_{1}-x_{2}})$$

**Figure 17:**
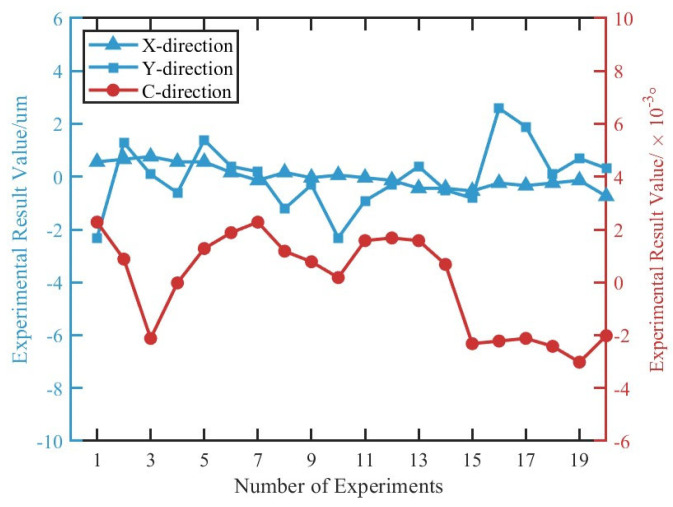
Results of visual positioning repeatability experiments.

In summary, the vision module enables the acquisition of the workpiece center coordinates and the *C*-axis rotation angle. The self-positioning system was tested over 20 repeated trials to further evaluate the positioning accuracy. As shown in Figure 17, the X-direction repeatability is 1.3 μm (3σ), the Y-direction repeatability is 3.7 μm (3σ), and the *C*-axis rotational repeatability is 0.005° (3σ), all of which meet the positioning accuracy requirements for MRF.

### 3.3. Probe Data Acquisition and Ball Tip Radius Compensation

**Figure 18:**
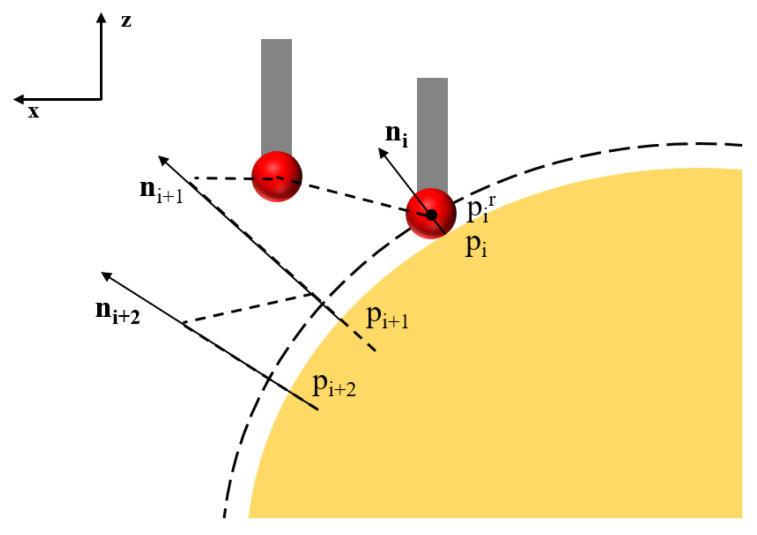
Schematic diagram of probe data acquisition.

A five-dimensional contact-trigger probe  (±X,±Y,+Z) is used to collect surface data along the normal direction, as illustrated in Figure 18. Since the probe records the coordinates of the center of the ball tip rather than the actual contact point, radius compensation is required. When the contact point is denoted as pi, the recorded coordinates can be expressed as [31]

(24) $$p_{i}^{r}=p_{i}\pm rn_{i}$$

(25) $$n_{i}=\frac{\left[1,-1/f\prime (x_{i},y_{i})\right]}{\sqrt{1+1/f\prime^{2}(x_{i},y_{i})}}$$

where r is the radius of the ball tip, and f′(x,y) is the surface normal vector, derived from the first-order derivatives of the surface equation.

Common compensation methods in coordinate metrology include the micro-plane method, micro-sphere method, and three-point circle fitting method [42]. In practical machining applications, where both the surface equation $f\left(x,y\right)\;$ and the probe radius are known, the equidistant surface method is adopted to compensate for the ball tip radius. For rotationally symmetric workpieces, a two-dimensional profile f(x) is used to approximate the three-dimensional surface $f\left(x,y\right)$. Let the equidistant surface function be denoted as $f_{r0}(x)$. Due to the analytical complexity of solving $f_{r0}(x)$ exactly, a polynomial function $f_{r}(x)$ is used to approximate the equidistant surface. Based on Equation (24), a sequence of sampling points on the equidistant surface is generated accordingly.

(26) $$P=[p_{1},p_{2},p_{3}\ldots p_{n}]$$

Let the polynomial equation be defined as

(27) $$f_{r}(x)=\theta_{1}x^{m}+\theta_{2}x^{m-1}\ldots +\theta_{m+1}$$

The polynomial order m is determined by the surface equation $f\left(x,y\right)$. By substituting the sampling point set P into the polynomial model, the coefficients $\theta =[\theta_{1},\theta_{2}\ldots \theta_{m+1}]$ can be obtained using the least-squares method.

**Figure 19:**
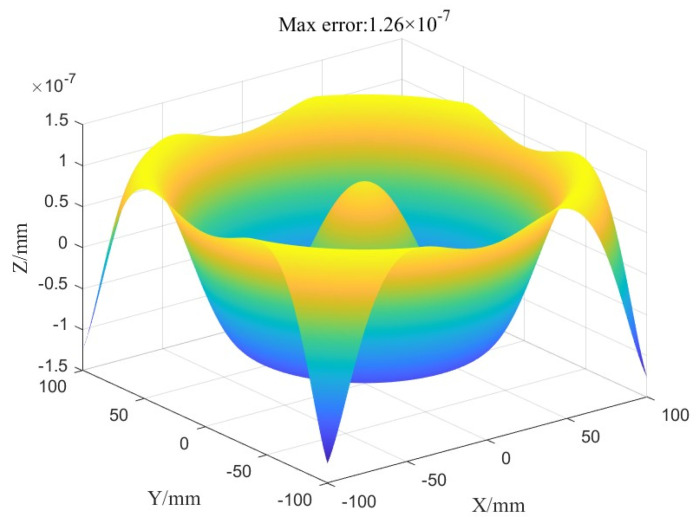
Polynomial fitting error of ball radius compensation.

Taking asphere #1 as an example (the specific parameters are shown in Table 4), and as shown in Figure 19, the fitting error distribution indicates a maximum error of 1.26 × 10^−7^ mm, which is significantly smaller than the machine tool positioning error of 3 μm and can be considered negligible.

### 3.4. Design of a Stepwise Global Optimization Algorithm

The essence of workpiece localization lies in determining the transformation relationship from the workpiece coordinate system O_W_ to the machine program coordinate system O_M_. Define the transformation as follows:

(28) T=G(g)

where g=[x,y,z,a,b,c] represent the translation and rotation parameters from the workpiece coordinate system O_W_ to the machine coordinate system O_M_. The function G(**g**) converts the vector g into a 4 × 4 homogeneous transformation matrix **T**.

The measured point coordinates of the workpiece in the machine coordinate system O_M_ are denoted as $p_{i}=[x_{i},y_{i},z_{i},1]\prime$, $P=[p_{1},p_{2}\ldots p_{n}]$ and the corresponding coordinates in the workpiece coordinate system O_W_ are denoted as $q_{i}=[x_{i}\prime ,y_{i}\prime ,z_{i}\prime ,1]\prime$, $Q=[q_{1},q_{2}\ldots q_{n}]$. The transformation relationship is given by

(29) $$Q=T^{-1}P$$

However, due to unavoidable workpiece shape deviations and measurement errors, Equation (29) does not hold exactly in practice. In self-positioning of the workpiece, the sum of squared distances is used as the objective function. The goal is to optimize both T and Q to minimize the error defined in Equation (30). Existing algorithms for this problem include the SVD algorithm, ICP (iterative closest point), and Tangent algorithm [43,44,45], which are typically solved using alternating iterative methods

(30) $$e(T,Q)=\sum_{i=1}^{n}∥T^{-1}p_{i}-q_{i}{∥}^{2}$$

Considering that the surface profile of the workpiece polished by magnetorheological processing is known, a previous study [31] proposed a synchronous optimization-based localization criterion. For a point on the surface defined as $q_{i}=[x_{i}\prime ,y_{i}\prime ,f(x_{i}\prime ,y_{i}\prime ),1]\prime$, the ideal measurement point after the rigid body transformation T is $p_{i}\prime$; then,

(31) $${\left[\begin{array}{cccc} x_{i}\prime & y_{i}\prime & f(x_{i}\prime ,y_{i}\prime ) & 1 \end{array}\right]}^{T}={\left[\begin{array}{cccc} \left(T^{-1}p_{i}\prime )(1\right) & \left(T^{-1}p_{i}\prime )(2\right) & \left(T^{-1}p_{i}\prime )(3\right) & 1 \end{array}\right]}^{T}$$

Then, the following condition holds [31]:

(32) $$(T^{-1}p_{i}\prime )(3)-f((T^{-1}p_{i}\prime )(1),(T^{-1}p_{i}\prime )(2))=0$$

Taking into account measurement noise and ball tip radius compensation, Equation (32) can be described as

(33) $$F(T)=(T^{-1}P)(3)-f_{r}(∥[(T^{-1}P)(1),(T^{-1}P)(2)]∥)$$

The localization objective function is then defined as

(34) $$\Psi (T)=\;∥F(T){∥}^{2}$$

**Figure 20:**
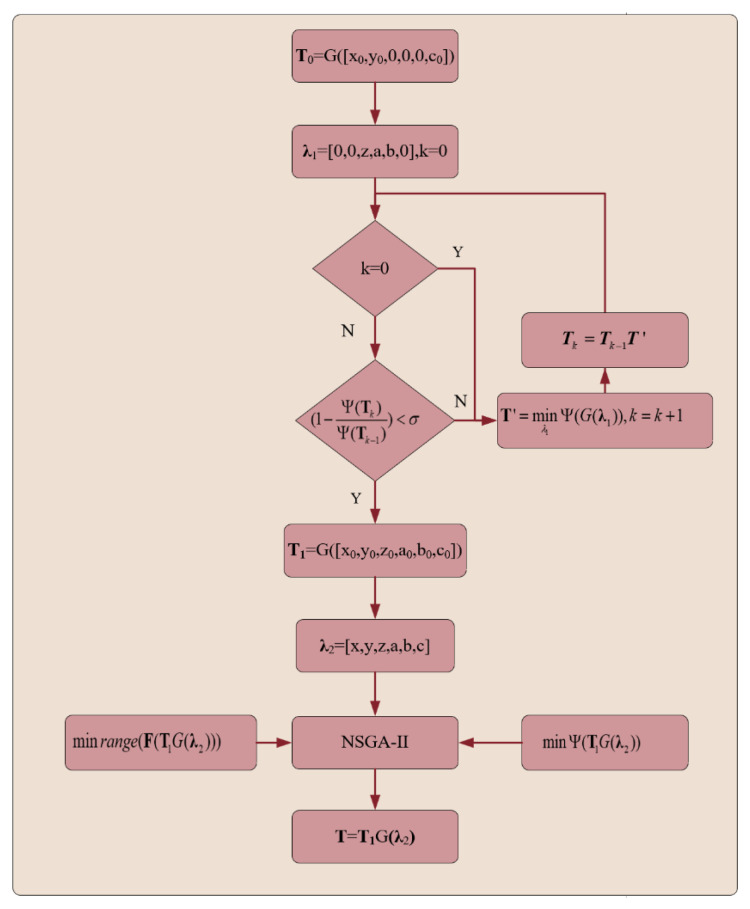
The stepwise global optimization algorithm.

A stepwise global optimization model is constructed based on the localization criterion, as illustrated in Figure 20. An initial transformation matrix **T**_0_ is established using the center coordinates $\left(x_{0},y_{0}\right)$ and *C*-axis rotation angle *c*_0_ obtained from the vision-based localization. In the first stage, the degrees of freedom [x,y,c] are fixed, and Equation (34) is used as the objective function to optimize the remaining degrees of freedom [z, a,b], yielding the rigid body transformation matrix **T**_1_. The resulting six-degree-of-freedom offset is $\left[x_{0},y_{0},z_{0},a_{0},b_{0},c_{0}\right]$. Due to measurement uncertainties, the obtained **T**_1_ may not represent the globally optimal transformation. To further improve accuracy, an additional criterion is introduced on top of Equation (34):

(35) $$range(F(T_{1}G(\lambda_{2})))$$

Specifically, the additional criterion is defined as the range (maximum deviation) of $F(T_{1}G(\lambda_{2}))$. Minimizing Equation (35) constraints $T^{-1}P$ to remain near the ideal surface, effectively preventing large deviations during the optimization process. The NSGA-II [46] algorithm is used to perform global optimization over all six degrees of freedom. By simultaneously minimizing Equations (34) and (35), the final pose transformation matrix T is obtained.

The feasibility of the proposed algorithm is verified through numerical simulation, and the surface shape used in the simulation is #1 aspherical surface. As shown in Table 5, the system noise during data acquisition is characterized. To enhance the credibility of the simulation, the measurement errors from both the probe and the vision-based localization are magnified by a factor of four. Specifically, random noise following normal distributions $N(0,0.01^{2})$ and $N(0,0.005^{2})$ is added to simulate the probe and vision errors, respectively.

**Figure 21:**
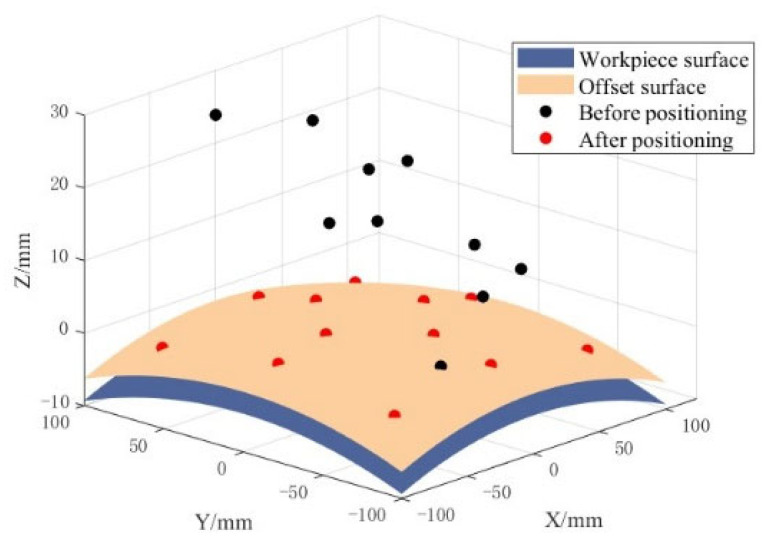
Simulation results of positioning algorithm.

The simulation results of the proposed algorithm are shown in Figure 21. From Table 6, it can be seen that after the first-stage optimization, the maximum error from the ideal transformation is 11 μm/0.003°. After global optimization, the error is further reduced to 6 μm/0.002°. The deviations in all six degrees of freedom meet the specified design criteria, demonstrating the feasibility and effectiveness of the proposed algorithm.

## 4. Experimental Validation and Results Analysis

The proposed method of workpiece self-positioning was verified through the surface error correction experiments on the plane and spherical workpieces. Due to the high cost of the experiments for aspherical workpieces, spherical workpieces were selected in this study to verify the applicability of the self-positioning method proposed for curved workpieces.

### 4.1. Experimental Platform and Test Conditions

**Figure 22:**
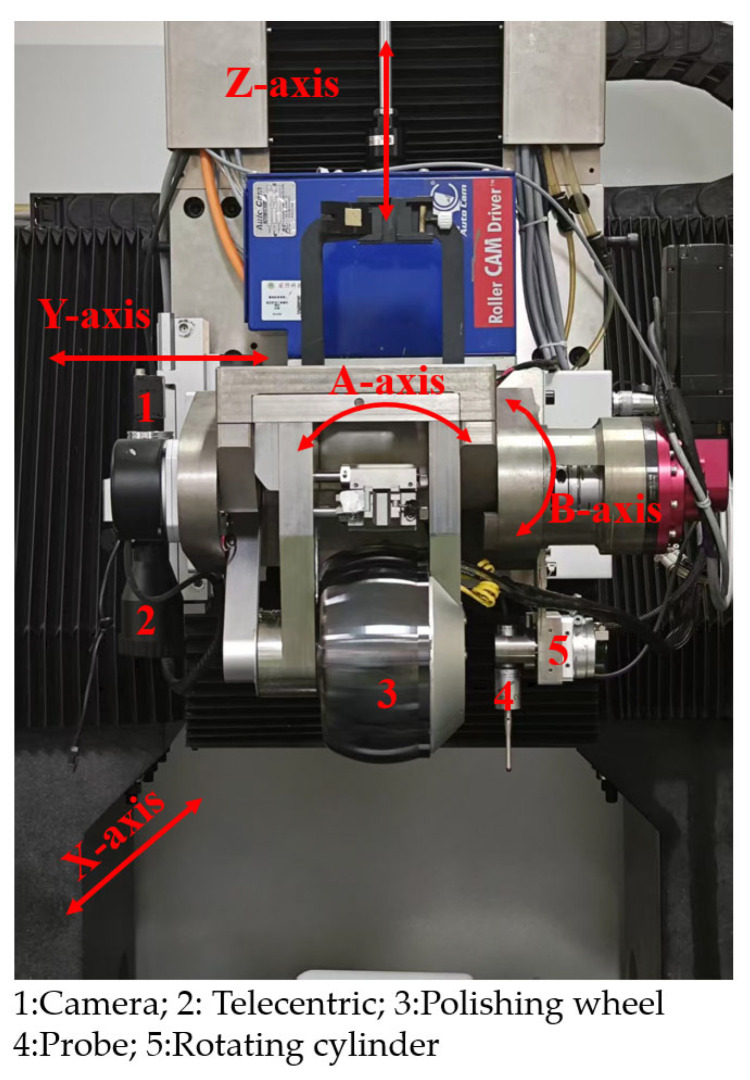
In-house-developed MRF machine.

**Figure 23:**
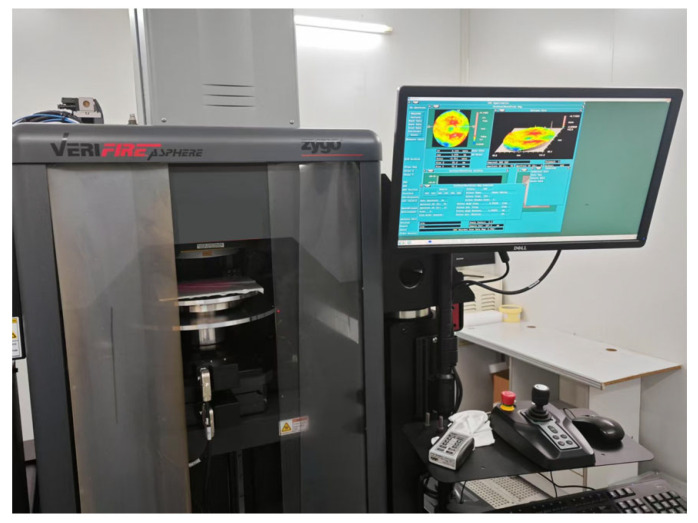
Zygo phase-shifting interferometer.

To evaluate the machining performance of the self-positioning method, it was integrated into an MRF machine developed in-house by the research group, as shown in Figure 22. A comparative experimental approach was adopted: workpieces were mounted using both the proposed self-positioning method and traditional manual alignment. Under identical processing parameters, correction experiments were conducted on both planar and curved workpieces. Detailed workpiece specifications are provided in Table 7. The surface accuracy was measured using a Zygo (Zygo Corporation, Middlefield, CT, USA) phase-shifting interferometer (Figure 23), and a convergence ratio within the 95% clear aperture was used as the evaluation metric.

### 4.2. Experimental Results

**Figure 24:**
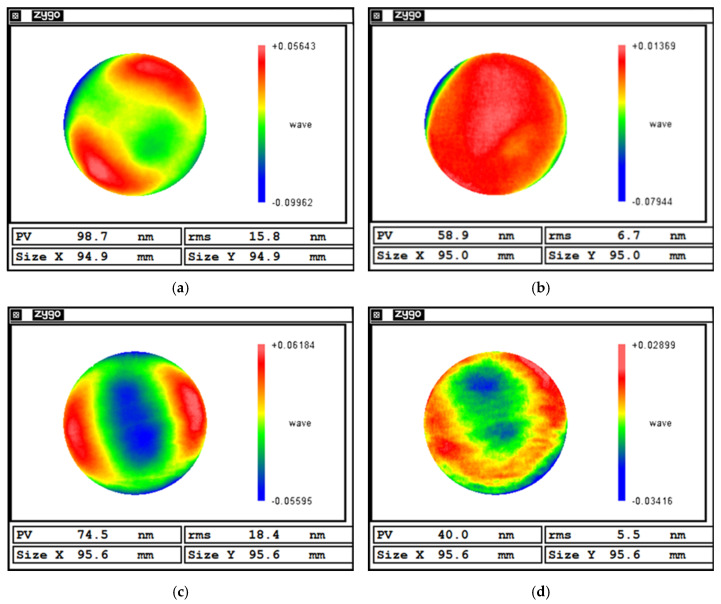
Planar surface correction results: (**a**) Before polishing with traditional alignment. (**b**) After polishing with traditional alignment. (**c**) Before polishing with self-positioning. (**d**) After polishing with self-positioning.

Figure 24b,d show the results of the surface figure error after polishing a plane workpiece. The polishing result of the self-positioning method is significantly better than that of the traditional alignment.

As shown in Table 8, under traditional alignment, the planar mirror achieved a PV convergence ratio of 1.68 and an RMS convergence ratio of 2.36. In contrast, using the proposed self-positioning method, the PV and RMS convergence ratios increased to 1.86 and 3.35, respectively. These results demonstrate that the self-positioning method meets the precision requirements for MRF and offers a superior convergence performance compared to traditional alignment. Moreover, in terms of the processing time, the self-positioning approach reduces the polishing time by 66.7% relative to manual alignment.

**Figure 25:**
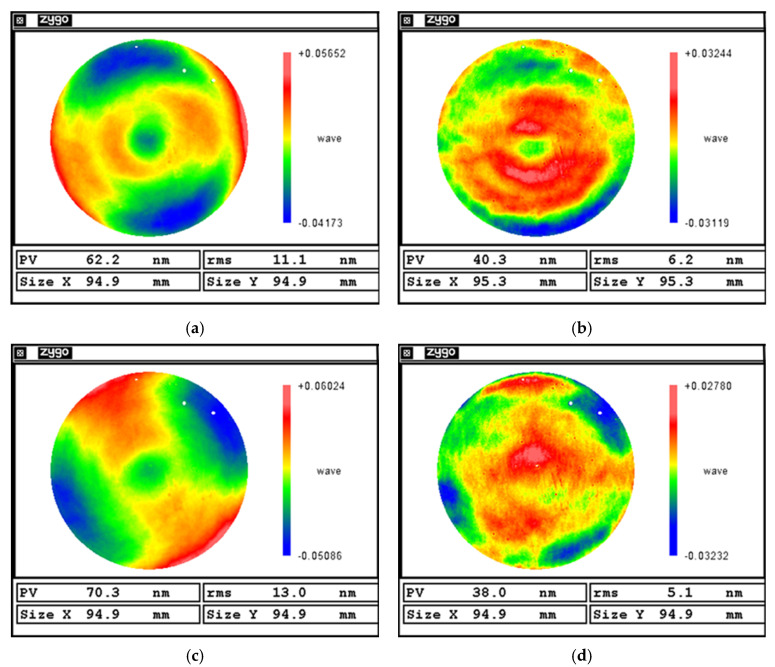
Curved surface correction results: (**a**) Before polishing with traditional alignment. (**b**) After polishing with traditional alignment. (**c**) Before polishing with self-positioning. (**d**) After polishing with self-positioning.

Figure 25b,d show the results of the surface figure error after polishing a spherical workpiece. Similar to the results of polishing flat workpieces, the polishing result of the self-positioning method is significantly better than that of the traditional alignment.

As shown in Table 9, the curved surface polishing results indicate that, after a single polishing cycle, the surface under traditional alignment improved from an RMS error of 11.1 nm to 6.2 nm. In comparison, the workpiece positioned using the self-positioning method achieved an RMS reduction from 13.0 nm to 5.1 nm, representing a 25.7% improvement in the convergence ratio. Additionally, the self-positioning method reduced the setup time by 80%, highlighting its efficiency and precision in curved surface correction.

## 5. Discussion and Conclusions

To address the limitation of convergence in single-pass polishing caused by workpiece positioning errors, this paper proposes a hybrid self-positioning method that integrates machine vision and probing technologies, which can effectively improve the polishing convergence ratio and reduce the workpiece loading and adjusting time. Firstly, this study systematically investigated the influence of positioning errors on the polishing convergence, and specifies the error tolerance threshold of each degree of freedom of the polishing convergence ratio. Secondly, a hybrid self-positioning method is proposed, and a stepwise global optimization model is constructed. Finally, the experiment verifies the effectiveness of the proposed method. However, the positioning error tolerance threshold derived in this paper is only applicable to small- and medium-caliber optical components (diameter less than 300 mm), and the proposed self-positioning method is suitable for rotationally symmetric optical components. Future research will focus on the positioning technology for non-regular workpieces. We believe that with the widespread application of high-efficiency and high-precision non-contact data acquisition methods, the alignment stage of magnetorheological polishing is moving towards automation and intelligence.

The main conclusions are as follows:

(1) A positioning error-normal contour error transmission model was established. Numerical simulations were conducted to analyze the impact of each degree of freedom on surface figure convergence. The results indicate that normal positioning errors have a far greater influence than tangential errors. Error tolerance thresholds were clearly defined: X/Y-direction errors ≤ 10 μm, Z-direction errors ≤ 5 μm, A/B-direction errors ≤ 0.005°, and C-direction errors ≤ 0.01°.
(2) The vision module, based on CNC servo feedback, resolves the trade-off between field of view and precision. By integrating peak detection, an improved Canny algorithm, and edge fitting techniques, the method achieves a repeatable localization accuracy better than 5 μm/0.005° in the X, Y, and C directions.
(3) The probe module collects 3D coordinate data, and polynomial-based equidistant surface fitting is used to compensate for the ball tip radius. A stepwise global optimization model is constructed by combining a synchronous iterative localization algorithm with the NSGA-II multi-objective optimization algorithm. The simulation results show that for curved workpieces, only nine measurement points (25 in reference [31]) are needed to achieve a positioning accuracy better than 10 μm/0.01°.
(4) The experimental results demonstrate that, compared to traditional alignment, the proposed self-positioning method improves the convergence ratio by 41.9% for planar workpieces and reduces the setup time by 66.7%. For the curved workpiece, the convergence ratio increases by 25.7%, with an 80% reduction in the alignment time.

In summary, the proposed self-positioning method satisfies the precision requirements of MRF [29,30], offering superior accuracy and stability over manual alignment. It enhances the automation level of polishing equipment and provides both theoretical and practical support for high-precision, high-efficiency polishing and intelligent optical manufacturing.

## Figures and Tables

**Table 1:** Experimental settings.

Parameter	Value
Polishing wheel speed (rpm)	200
Magnetic field current (A)	6.5
Flow rate (L/h)	100
Indentation depth (mm)	0.18/0.2/0.22/0.23/0.24/0.245/0.250/0.255/0.260/0.27/0.28/0.3/0.32

**Table 2:** Positioning error settings.

Direction	Magnitude										
X/mm	0.005	0.01	0.015	0.02	0.025	0.03	0.04	0.05	0.06	0.08	0.1
Z/mm	0.005	0.01	0.015	0.02	0.025	0.03	0.04	0.05	0.06	0.08	0.1
A/°	0.005	0.01	0.015	0.04	0.05	0.06	0.08	0.1	0.12	0.16	0.2
Combined	X = 0.01 mm, Y = 0.01 mm, Z = 0.005 mm, A = 0.005°, B = 0.005°										

**Table 3:** Key component performance parameters.

Component	Specification
Camera (A3B00MG000, iRAYPLE, Hangzhou, China)	Resolution: 5472 × 3648; pixel size: 2.4 μm
Lens (CR-XF-10MDT05X220D-1C, Shenzhen Can-Rill Technologies, Shenzhen, China)	Focal length: 220 mm; optical magnification: 0.5×
Cylinder (FESTO-DSM-12-270-P-A-B, Festo AG & Co. KG, Esslingen, Germany)	Working pressure: 0.2–1 MPa
Probe (Renishaw-LP2, RENISHAW, Wotton Under Edge, UK)	Trigger force: 5.85 N; repeatability: 1 μm

**Table 4:** Aspheric parameters.

Parameter Name	Value
Aperture (mm)	100
R(mm)	1065.36
K	−2.18

**Table 5:** System repeatability.

Category		Combined Error (σ)
Probe data acquisition	Machine tool motion error (3 μm)	2.45 um
	Probe triggering error (1.5 μm)	
	Workpiece form error (10 μm)	
Vision-based localization error		1.23 um

**Table 6:** Simulation results of the algorithm (unit: mm and °).

Offset Setting	g = [50, 10, 8, 10, 6, 0]	|g − g| = [0, 0, 0, 0, 0, 0]
After initial optimization	g_1_ = [49.989, 10.007, 8.002, 10.003, 6.001, 0]	|g − g_1_| = [0.011, 0.007, 0.002, 0.003, 0.001, 0]
After global optimization	g_2_ = [49.999, 10.006, 8.003, 10.001, 6.002, 0]	|g − g_2_| = [0.001, 0.006, 0.003, 0.001, 0.002, 0]

**Table 7:** Workpiece specifications.

Type	Aperture (mm)	Radius of Curvature (mm)	Material	Mechanical Properties of Materials
Planar	100		Fused Silica	Mohs hardness: 7 Thermal expansion coefficient: 0.5 × 10^−6^/°C Dense amorphous SiO_2_ structure
Spherical	100	400		

**Table 8:** Planar surface correction data.

	Before Polishing		After Polishing		Convergence Ratio		Time/min
	PV/nm	RMS/nm	PV/nm	RMS/nm	PV/nm	RMS/nm	
Traditional alignment	98.7	15.8	58.9	6.7	1.68	2.36	~30
Self-positioning	74.5	18.4	40.0	5.5	1.86	3.35	~10

**Table 9:** Curved surface correction data.

	Before Polishing		After Polishing		Convergence Ratio		Time/min
	PV/nm	RMS/nm	PV/nm	RMS/nm	PV/nm	RMS/nm	
Traditional alignment	62.2	11.1	40.3	6.2	1.54	1.79	~50
Self-positioning	70.3	13.0	38.0	5.1	1.85	2.55	~10

## Data Availability

The data presented in this study are available upon request from the corresponding author. The data are not publicly available because they are part of an ongoing study.
